# Quantum Capacity of Continuously Observed Ion Channels

**DOI:** 10.3390/e28050555

**Published:** 2026-05-15

**Authors:** Paulina Trybek, Jerzy Dajka

**Affiliations:** Institute of Physics, University of Silesia in Katowice, 75. Pułku Piechoty 1, 41-500 Chorzów, Poland; paulina.trybek@us.edu.pl

**Keywords:** ion channels, quantum capacity, continuous quantum measurement

## Abstract

A quantum model describing ion channels from an information-theoretic perspective is considered. The information χ-capacity of an ion channel, treated as an information channel whose properties are modified by continuous quantum measurements, is investigated. The behavior of the χ-capacity is analyzed as a function of the measurement parameters, in particular the type of measured observable, the measurement duration, and the measurement strength. It is shown that the information χ-capacity exhibits qualitatively different behaviors depending on the measurement conditions, including regimes of rapid decay as well as regimes where it remains finite for long observation times. These results indicate that, within the considered model, continuous observation may significantly influence the information-theoretic properties of the effective ion-channel dynamics.

## 1. Introduction

Ion channels are highly efficient molecular systems responsible for the selective transport of ions across cell membranes. Their activity is typically characterized in terms of conductance, which reflects the rate at which ions pass through the channel and depends on both the structural and energetic properties of the pore. These systems are generally described by three main properties: their permeability to ions, their ability to discriminate between different ionic species with similar or different charges, and their regulated gating, which controls the transition between open and closed states in response to specific stimuli [1,2]. Together, these mechanisms determine their functional role in cellular signaling and membrane transport.

Although ion channels have been extensively studied across multiple disciplines, the detailed mechanism of ion permeation remains an open and actively investigated problem. In particular, understanding how ion selectivity emerges within the narrow selectivity filter, whose width is only a few angstroms, continues to pose significant theoretical and experimental challenges. This property, defined as the ability of a channel to preferentially conduct specific ions, has been addressed using a variety of approaches, including thermodynamic analyses, structural models of the pore, and statistical theories of selectivity or conductance [3,4,5,6].

Current research on ion channels is primarily aimed at uncovering the molecular mechanisms that govern their fundamental properties and modulation by ligands and other regulatory factors. Theoretical and computational frameworks have been widely used to describe key processes such as permeation, selectivity, and gating. With the continued development of computational methods and growing computational capabilities, progressively refined models have been proposed that incorporate additional factors, including channel geometry [7], ion–ion and ion–environment interactions [8], and hydrophobic gating effects in ion transport [9].

While classical approaches typically describe channel function in terms of ion flux and conductance, some recent theoretical frameworks adopt a broader description of ion transport as a dynamical process that can be interpreted in terms of information processing at the level of channel states and ionic configurations. This interpretation is particularly natural in systems where transport proceeds through a discrete set of metastable configurations, as is the case for ions occupying binding sites within the selectivity filter. Within this viewpoint, the channel is treated not only as a passive conduit for ions but also as a system that encodes information through its transport dynamics. This broader perspective has motivated a range of theoretical models, including approaches that incorporate quantum-mechanical features [10,11].

The application of quantum theory to ion channels has been explored in a growing body of literature. Kariev et al. emphasized that key processes occurring in ion channels, including charge transfer, may require a quantum-mechanical description [12]. Other studies have investigated the role of quantum effects in ion transport [13]. In particular, Salari et al. [14] proposed that quantum interference may contribute to ion selectivity mechanisms, whereas Vaziri et al. [15] suggested that quantum coherence within the selectivity filter could influence both ion conduction and specificity.

A recent quantum-mechanical description of ion transport was proposed by Seifi et al. in Ref. [16], where the authors introduced a minimal three-state model of the selectivity filter and explored the role of quantum coherence in the corresponding transport processes. The considered configurations can be naturally related to metastable occupancy states within the selectivity filter, commonly described in terms of binding sites such as S1–S4 in potassium channels like KcsA [17]. These correspond to discrete ion-binding positions along the filter. The resulting configurations define an effective state space that can be interpreted in terms of information encoding within the selectivity filter, where different occupancy patterns correspond to distinguishable system states. In biological ion channels, efficient conduction is believed to rely on coordinated multi-ion motion within the selectivity filter, often described in terms of knock-on mechanisms involving alternating ion–water configurations. Within this picture, transport is governed not only by individual ion transitions but also by correlations between different occupancy states. Quantum coherence, as introduced in reduced models of ion channels, may influence the formation and stability of such correlated configurations. Building on this framework, Seifi et al. further analyzed how quantum coherence is related to the hopping rate, defined as the transition rate at which ions overcome energy barriers between distinct states during the conduction process.

Although phenomenological in nature, this approach provides a useful framework for discussing nontrivial aspects of ion transport within a quantum formalism. However, such reduced descriptions do not explicitly resolve the role of environmental interactions and measurement-like effects, which are inherent to biological ion channels.

Using the three-state framework introduced above, the present work considers the dynamics of an ion channel under continuous measurement of selected observables. The ion channel is treated as a communication channel, where the system states encode information, while continuous non-selective measurements act as a source of noise affecting its transmission. Both global properties of the system and projections onto individual states are considered, which can be interpreted as monitoring ionic configurations within the selectivity filter. This measurement-induced modification of the dynamics leads to an effective quantum channel description.

The information-processing capabilities of the system are then analyzed using the Holevo capacity introduced in [18], which quantifies the maximal amount of information that can be reliably transmitted despite the presence of noise. From a physical standpoint, the χ-capacity can be interpreted as a measure of how efficiently coherent ionic configurations encode and transmit information through the selectivity filter. Its reduction therefore reflects not only the loss of abstract quantum information, but also the suppression of correlations underlying coordinated ion transport. In this framework, ion transport can be viewed not only as a physical process but also as information transmission under noise, allowing one to assess to what extent it retains genuinely quantum features under continuous observation. As we will demonstrate, this formulation provides a complementary viewpoint, especially in the presence of measurement, which in quantum models alters the information content of the system. Environmental interactions or measurement-like processes introduce noise, thereby affecting information transmission. Consequently, concepts from quantum information theory, such as channel capacity, offer a natural way to quantify how much information about the system’s state can be preserved during ion transport. This approach thus provides a bridge between microscopic descriptions of ion dynamics and information-theoretic measures of transport efficiency, and is made explicit here by considering continuous non-selective measurements of selected observables, which induce a measurement-dependent quantum channel. The resulting information transmission properties are quantified numerically via the Holevo χ-capacity as a function of the measurement strength σ, evolution time *t*, and the Hamiltonian parameter *c*. This description provides complementary information to standard transport characteristics, including conductance or ion flux. In particular, while conventional approaches quantify the rate of ion transport, the information-theoretic framework captures the structure of the underlying dynamics, including how transitions between different ionic configurations are organized and how robust this structure remains under environmental perturbations. In this sense, the χ-capacity allows one to distinguish between regimes in which transport proceeds via well-defined, structured transitions between configurations and those in which environmental interactions lead to effectively randomized dynamics. This provides a way to characterize the degree of organization of the transport process, which is not directly accessible through traditional observables.

We organize this paper as follows. The section *Methods* begins with a concise overview of the phenomenological three-state ion channel model introduced in Ref. [16]. We then introduce the communication-theoretic framework used to analyze the system, representing the measurement-induced dynamics in terms of quantum channels. The *Results* section presents numerical simulations of the resulting dynamics, along with their analysis and interpretation in terms of ion transport through the selectivity filter. Finally, we summarize the main findings and discuss possible directions for future research.

## 2. Materials and Methods

**Figure 1:**
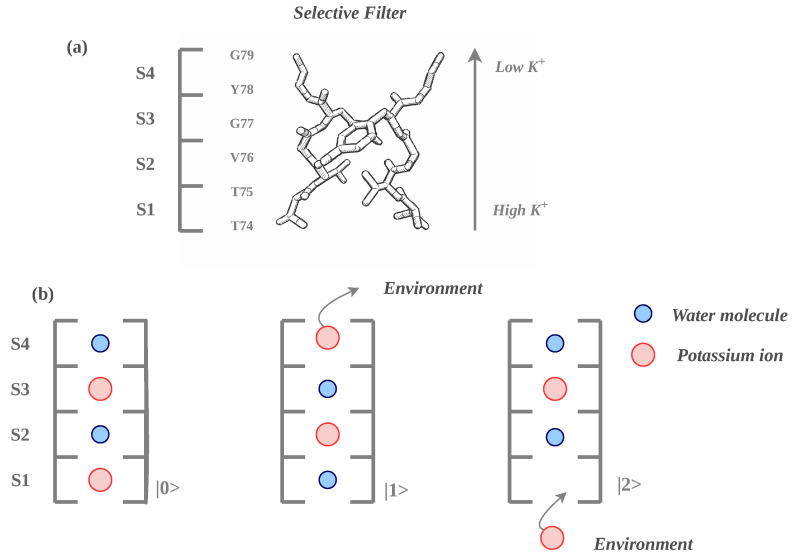
(**a**) Representation of the selectivity filter of the KcsA ion channel (based on *PDB 1K4C*, visualized in Jmol) with four symbolic K^+^ ion-binding sites (S1–S4), defined according to the convention adopted in Ref. [16], together with the corresponding amino-acid arrangement; (**b**) Schematic illustration of the ion-channel state configuration: |0〉 (left), |1〉 (middle), |2〉 (right).

The phenomenological quantum model introduced in Ref. [16] and employed in the present work describes the selectivity filter region as an effective four-site channel, where ion transport is reduced to a small set of dominant configurations. Rather than resolving all microscopic details of the permeation process, the model focuses on three effective states that capture the essential features of the coordinated motion of ions and water molecules during translocation. The graphical representation of the model is presented in Figure 1.

In this reduced description, the different configurations correspond to distinct occupancy patterns within the selectivity filter, associated with the so-called knock-on permeation mechanism (In the direct knock-on mechanism, K^+^ ions traverse the selectivity filter in a dehydrated state, making direct ion–ion contacts within the conserved filter architecture. By contrast, the soft knock-on mechanism proposes that water molecules are present in the filter and co-permeate with K^+^ ions, maintaining separation between adjacent ions during conduction [19]). The effective states can be interpreted as different arrangements of ions and water molecules distributed across the binding sites (S1–S4), including configurations in which ions occupy alternating positions or are exchanged with water molecules along the filter.

The first state, denoted as |0〉 (see Figure 1, panel b), corresponds to a configuration in which two K^+^ ions occupy alternating sites within the filter (e.g., S1 and S3), while the remaining sites are filled with water molecules. This arrangement reflects a typical configuration in which ions are separated by water molecules, consistent with the soft knock-on picture of permeation. The second state, |1〉, represents the complementary alternating configuration (e.g., ions at S2 and S4), effectively describing a shifted arrangement along the filter axis. Transitions between |0〉 and |1〉 capture the concerted motion of ions and water molecules along the selectivity filter, consistent with the knock-on mechanism. The third state, |2〉, accounts for the exchange process at the channel boundary and represents a configuration in which an ion has been released from the filter to the extracellular (or intracellular) side and a new ion enters the filter. In this sense, |2〉 effectively incorporates the coupling of the selectivity filter to the surrounding ionic reservoirs and enables a cyclic description of the conduction process.

Transitions between these configurations are driven by an effective energy landscape dominated by electrostatic interactions of Coulomb type [5]. Within the quantum formulation of Ref. [16], the system is represented in a three-dimensional Hilbert space spanned by basis states |i〉, i=0,1,2, each corresponding to one of the effective configurations described above.

The dynamics are generated by the Hamiltonian

(1) $$H_{sys}=H_{0}+cV,\;H_{0}=S_{z},\;V=\sum_{i=0}^{2}\left|\left(i+1\right)mod\;3\rangle \langle i\right|$$

where *c* denotes the coherent hopping amplitude, while the operator *V* describes coherent transitions between the configurations associated with the knock-on permeation mechanism. This structure reflects the fact that the effective states form a closed dynamical cycle, allowing for a continuous sequence of ion translocation events. It should be emphasized that the model used is probably the simplest model capable of describing the most important quantum properties potentially characterizing ion channels from a mathematical and physical perspective. It should be noted that such a simplified description may in many cases constitute an oversimplification and is not intended to capture phenomena related, for example, to the microscopic structure of ion channels. The type of modeling employed here is classified as a “soft knock-on” mechanism, which, unlike the “hard knock-on” picture based primarily on short-range ion–ion interactions, emphasizes the role of electrostatic interactions involving both ions and water molecules [20].

A continuous measurement [21] involves the continual extraction of information from a system, whereas a non-selective measurement is one where the result is ignored or not read [22]. The first part of the measurement is described by the system-probe total Hamiltonian:

(2) $$\begin{array}{c} H_{sys+probe}=H_{sys}+H_{probe}+g\left(t\right)H_{int} \end{array}$$

where g(t) is the strength of the coupling. The observable *A* of a quantum system couples to the probe’s position *Q* resulting in the interaction $H_{int}=A⊗Q$.

Assuming a non-selective measurement performed over a time interval divided into sub-intervals of length *q*, the dynamics of the system, after being traced with respect to the probe degrees of freedom, is no longer unitary but completely positive [22,23,24]. For a set of measurement operators $E_{x}$, where $\sum_{x}{E^{†}}_{x}E_{x}=I$, the non-selective measurement of a system in state ρ is the transformation $\rho \to E_{x}\rho {E^{†}}_{x},$ which is an example of a completely positive map [24]. The evolution of the state due to such a measurement on each time interval of length *q* is given by [21,22]:

(3) $$\begin{array}{c} \rho \left(t+q\right)=\int dpE_{p}\rho \left(t\right)E^{†}p \end{array}$$

with $E_{p}=e^{iqHsys}\langle p|e^{-iqH_{int}}|\phi_{q}\rangle .$ Here, $|p\rangle \in H_{probe}$ is an eigenstate of *P*, the probe momentum, and $|\phi_{q}\rangle \in H_{probe}$ is the state of the probe before the measurement. Expanding Equation (3) to second order in *q* for a finite measurement duration leads to a quantum master equation:

(4) $$\begin{array}{c} \frac{d}{dt}\rho \left(t\right)=-i\left[H_{sys},\rho \left(t\right)\right]-\frac{\sigma }{2}\left[A,\left[A,\rho \left(t\right)\right]\right] \end{array}$$

where the dissipative term represents the back-action on the state of the system due to the measurement [23,24]. The parameter σ describes the back-action of the measurement. For an integrated coupling strength proportional to the length of the time interval $q∼\int_{0}^{t}g\left(s\right)ds$, σ is given by: $\sigma^{2}=\lim_{q\to 0}q\langle \phi_{q}|Q^{2}|\phi_{q}\rangle$. The parameter σ has a natural physical interpretation resulting from the quantum nature of the measurement performed, the ‘side effect’ of which, apart from obtaining information about the system under investigation, is the loss of coherence, and therefore the ‘classicization’ of the system under investigation. The larger the amplitude σ, the more the measurement-induced decoherence affects the dynamic properties of the system.

Here we consider continuous nonselective measurement of four observables: $A=S_{Z}$ and $A=P_{i}$ where $P_{i}=\left|i\rangle \langle i\right|$ denote projectors on three basis states of the ionic channel i=0,1,2. The choice of observables is natural. The measurement task is to observe particles passing through the channel at various characteristic points. The choice of the specific ion channel configuration to be measured determines the choice of observables. The specific measurement used may, for various technical reasons, prefer to observe one of the three basis configurations corresponding to the projection operators onto the appropriate states. The choice of the $S_{Z}$ observables effectively measures the expectation value of the Hamiltonian $H_{0}$, and therefore the energy of the undisturbed system. The dynamics of continuously and nonselectively measured ionic channels represented by solutions of Equation (4) form complete positive maps Φ or, in other words, quantum communication channels [25]. In particular, basic communication schemes consist of a classical-quantum channel (c-q) coding classical information *i* into a composition of *n* quantum states followed by a fully quantum channel (usually given by a completely positive map) and decoding into a classical output via a quantum-classical channel (q-c). These steps formally read as follows:

(5) $$i\overset{c-q}{\to }\rho_{i}^{\left(n\right)}\overset{q-q}{\to }\Phi^{⊗n}\left[\rho_{i}^{\left(n\right)}\right]\overset{q-c}{\to }j$$

A quantity that naturally characterizes communication channels is their capacity. In the case of quantum channels one defines the χ-capacity (Holevo capacity) [25] as follows:

(6) $$C_{\chi }\left(\Phi \right)=\underset{\left\{p_{i}\rho_{i}\right\}}{\sup}\left[S\left(\sum_{i}p_{i}\Phi \left[\rho_{i}\right]\right)-\sum_{i}p_{i}S\left(\Phi \left[\rho_{i}\right]\right)\right]$$

where S(ρ)=−Tr[ρlog(ρ)] in the celebrated quantum von Neumann entropy [23,24,25] and the supremum is calculated with respect to all probability distributions $p_{i}$ and states $\rho_{i}$. The χ-capacity Equation (6) is related to a classical capacity of a channel Φ in Equation (5) via:

(7) $$C\left(\Phi \right)=\lim_{n\to \infty }\frac{1}{n}C_{\chi }\left(\Phi^{⊗n}\right)$$

The χ-capacity formula Equation (6) has a natural interpretation that describes the quantum component of information encoded in a quantum manner via c-q channel and then transmitted through a q-q channel in terms of completely positive map Φ in our case given by Equation (4). In other words, calculating the capacity of the communication channel allows us to estimate to what extent ion channels subjected to the process of continuous measurement over time retain the quantum character of the information that is transmitted. It should be emphasized that the use of quantum measures of ion channel capacity, treated as information channels, allows us to expand their description with an information component related to the potential quantum correlations between ions existing within the channels. Taking this component into account allows us to expand the biophysical characterization of channels with essentially quantum features and to make their occurrence dependent on selected channel parameters and, as we do in this paper, on the type of measurement performed.

## 3. Results

This chapter presents numerical results for the χ-capacity of an ion channel as defined in Equation (6). The calculations are performed using QuTiP (version 5.2.3) [26,27,28], and the supremum in Equation (6) is evaluated over 1000 randomly generated initial states. It should be noted that the optimization procedure performed here, based on a set of random initial conditions, is heuristic in nature. It should be emphasized, however, that selecting a sufficiently large family of initial conditions, in our case three orders of magnitude larger than the minimum ensemble containing at least $d^{2}$ states (with *d* denoting the dimension of the state space) required for full optimization [25], allows the qualitative characteristics of the capacity to be reproduced, in particular its monotonicity and asymptotic behavior.

In the absence of measurement (σ=0), the system evolves coherently according to Equation (1) and retains its maximal χ-capacity, which serves as a reference point for the results discussed below. We have verified that the qualitative behavior of the results is robust with respect to the choice of initial states. We consider the χ-capacity of ion channels as a function of three factors: the measurement strength σ, the measurement duration *t*, and the coherent hopping amplitude *c* appearing in Equation (1), which characterizes the intrinsic transport properties of the channel.

**Figure 2:**
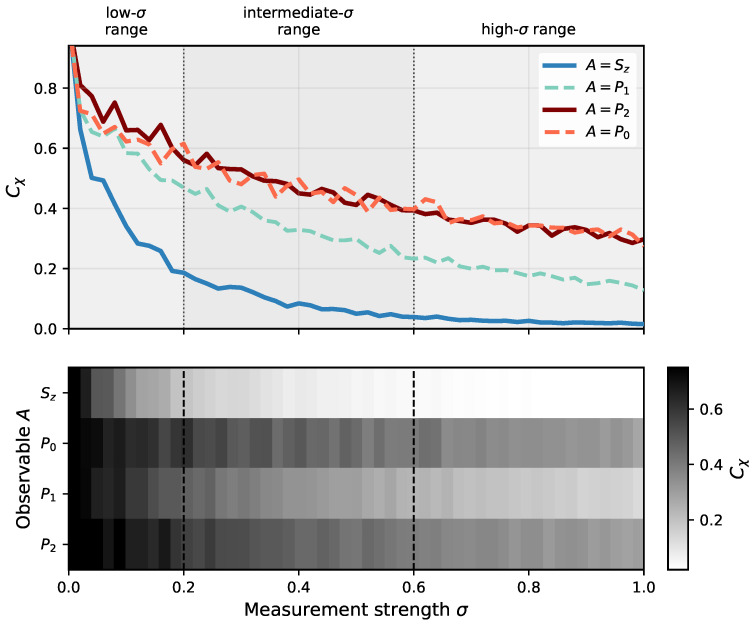
The χ-capacity, Equation (6), of an ion channel under continuous nonselective measurement of an observable *A* as a function of the measurement strength σ, calculated at t=100 and c=0.1 (cf. Equation (1)). The upper panel shows the corresponding line plots for different observables, while the lower panel presents a heatmap representation of the same data, providing a complementary view of the (σ,A) parameter space. The shaded regions serve only as a visual guide, indicating approximate ranges of σ associated with different qualitative behaviors of the χ-capacity.

The dependence on the measurement strength σ is presented in Figure 2, where the χ-capacity is shown for a fixed measurement duration t=100, corresponding to a time scale two orders of magnitude larger than the characteristic system time (in units where ℏ=1). As expected, the capacity decreases with increasing σ, reflecting the progressive suppression of quantum coherence due to measurement-induced decoherence. A clear distinction emerges between different observables. In particular, for $A=S_{z}$ and $A=P_{1}$, the χ-capacity decays rapidly, whereas for $A=P_{0}$ and $A=P_{2}$ the decay becomes significantly slower and the capacity remains finite over long times. This indicates that, although strong measurements generally reduce the information capacity, certain configurations retain a non-zero ability to encode quantum information even in the strong-measurement regime. This separation into two distinct classes of observables suggests that measurements associated with certain channel configurations (notably $P_{1}$) disturb the coherent dynamics more strongly than others, possibly due to their role in the cyclic transitions generated by the Hamiltonian. The projector $P_{1}$ (see state |1〉 in Figure 2), associated with intermediate configurations in the knock-on permeation process, corresponds to stages where the dynamics involves stronger temporal correlations and more pronounced coherent evolution. As a result, these configurations are more sensitive to measurement-induced decoherence, which leads to a rapid suppression of the χ-capacity. In contrast, the projectors $P_{0}$ and $P_{2}$, which can be associated with initial and final stages of the permeation cycle, correspond to configurations with weaker temporal correlations and are less disruptive under measurement, resulting in a more robust dynamical behavior. The comparatively slower decay of the χ-capacity for these observables suggests that these configurations are less affected by measurement and effectively form more robust dynamical sectors that retain a greater information-carrying capability over long times. Within the considered model, this separation may be interpreted as reflecting different roles of the effective configurations within the selectivity filter. Intermediate states associated with coordinated ion motion appear to depend more strongly on coherent dynamics and therefore become more sensitive to environmental perturbations, whereas configurations related to ion entry or release remain comparatively less affected by decoherence. This suggests that environmental noise does not influence all configurations uniformly, but may affect more strongly those states that are most closely connected with coherent transport processes.

**Figure 3:**
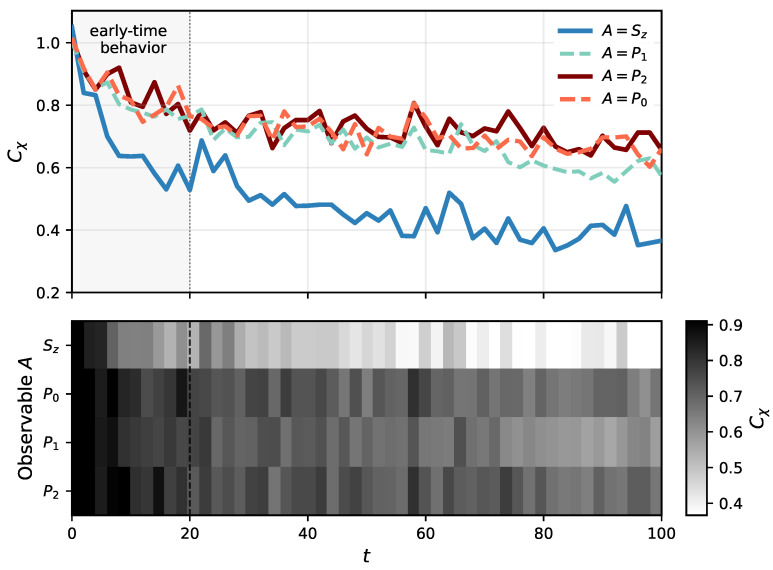
The χ-capacity, Equation (6), of an ion channel under continuous nonselective measurement of an observable *A* as a function of time *t*, calculated for σ=0.1 and c=0.1 (cf. Equation (1)). The upper panel shows the corresponding time dependence for different observables, while the lower panel presents a heatmap representation of the same data, providing a complementary view of the (t,A) parameter space. The shaded region highlights the early-time behavior of the dynamics.

A similar analysis of the dependence of the χ-capacity on the measurement time *t* is presented in Figure 3. The dynamics exhibit a two-stage behavior: an initial rapid decay followed by a slower evolution with visible fluctuations. As in the previous case, the fastest decay is observed for $A=S_{z}$. For the remaining observables $A=P_{i}$, i=0,1,2, the differences are less pronounced. In contrast to the dependence on σ, the χ-capacity exhibits a more persistent and slowly varying behavior in the long-time regime. This indicates that continuous nonselective measurement does not fully suppress coherent dynamics. Rather, the system remains in a dynamically evolving regime resulting from the interplay between Hamiltonian evolution and measurement-induced decoherence, in which a non-zero amount of quantum information can still be transmitted. In the context of ion-channel dynamics, this behavior can be associated with the persistence of a dynamically stable conduction regime in the selectivity filter, where the system operates under continuous interaction with its environment. The comparatively slow temporal decay of the χ-capacity suggests that the underlying ion configurations retain a degree of coordinated dynamical behavior over extended timescales, enabling transport even in the presence of environmental perturbations. Interestingly, at longer observation times, the intermediate configuration |1〉 associated with $P_{1}$ becomes more clearly separated from $P_{0}$ and $P_{2}$ (|0〉 and |2〉). This behavior may be related to the role attributed to the intermediate state within the considered knock-on permeation picture. The state represented by $P_{1}$ is associated with a transient stage of coordinated ion rearrangement within the selectivity filter, in contrast to the more stable configurations linked to ion entry or release. Since ion permeation involves such collective transitions between configurations, the intermediate state appears to depend more strongly on coherent dynamics and therefore becomes more sensitive to the long-time interplay between measurement-induced decoherence and Hamiltonian evolution.

**Figure 4:**
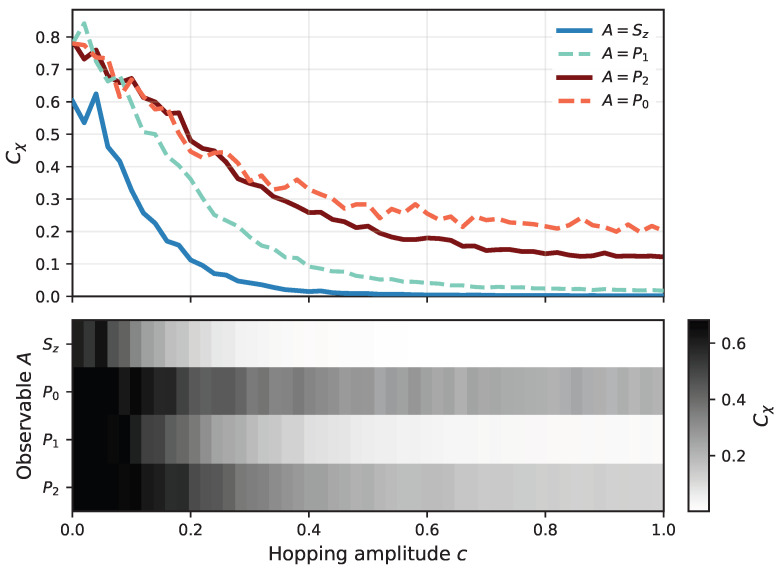
The χ-capacity, Equation (6), of an ion channel under continuous nonselective measurement of an observable *A* as a function of the coherent hopping amplitude *c*, calculated for t=100 and σ=0.1 (cf. Equation (1)). The upper panel shows the corresponding dependence for different observables, while the lower panel presents a heatmap representation of the same data in the (c,A) parameter space.

In Figure 4, we additionally analyze the behavior of the χ-capacity as a function of the coherent hopping amplitude *c*. The most pronounced decrease in the χ-capacity occurs in the regime of small *c*, where coherent transport between channel configurations is weak. As *c* increases, coherent transitions become more efficient, partially counteracting the decohering effect of continuous measurement. Similarly to the case of varying σ, we observe that the observables $A=S_{z}$ and $A=P_{1}$ lead to a significantly faster decay of the χ-capacity than $A=P_{0}$ and $A=P_{2}$. This again highlights the crucial role of the measured observable in determining the robustness of quantum information transmission through the channel.

Taken together, the results demonstrate that the degradation of the χ-capacity in continuously monitored ion channels depends not only on the strength and duration of the measurement, but also strongly on the nature of the observable and the intrinsic coherent dynamics governed by the Hamiltonian. Observables that interfere more directly with coherent transitions induce stronger decoherence and lead to a faster loss of the quantum component of the transmitted information.

Within the considered ion-channel model, the hopping amplitude *c* may be associated with the efficiency of transitions between effective configurations of the selectivity filter. Small values of *c* correspond to weaker coupling between configurations and less efficient transport dynamics, while larger values of *c* are associated with more coordinated and dynamically coherent ion motion. In this context, the different observables may be related to specific effective configurations of the filter. In particular, the projector $P_{1}$, associated with intermediate ion configurations within the channel (|1〉), probes states linked to transient stages of coordinated ion rearrangement. As a result, measurements associated with $P_{1}$ lead to a faster decay of the χ-capacity, indicating that these configurations are more sensitive to the interplay between coherent dynamics and measurement-induced decoherence. In contrast, the observables $P_{0}$ and $P_{2}$ (|0〉, |2〉), which may be associated with initial and final stages of the permeation cycle, remain comparatively less affected by the measurement process and exhibit a more persistent information-carrying capability. These observations suggest that the persistence of coherent transport-related dynamics in the model is closely connected with the role of specific configurations within the transport cycle and may therefore depend on structural or environmental factors influencing ion coordination within the selectivity filter. This interpretation remains consistent with previous studies of soft knock-on transport models, where coherent ion transitions and environmental interactions were found to jointly influence the transport properties of the channel [20].

## 4. Discussion

Systems exhibiting quantum properties require special attention in the context of measurement, since the act of observation inevitably affects their dynamics. Motivated by the possible quantum aspects of ion channel transport, we investigated the influence of continuous nonselective measurement of selected observables on the dynamics of the three-state ion channel model introduced in Ref. [16]. Rather than focusing exclusively on standard transport characteristics, we analyzed the corresponding information-theoretic properties of the model, in particular the behavior of the Holevo χ-capacity as a function of the selected system parameters.

In our study, we investigated how the χ-capacity changes with the measurement strength σ, the evolution time, and the hopping amplitude *c* encoding the effective ion-channel dynamics in the Hamiltonian. Numerical results revealed qualitatively distinct behaviors when different parameters were varied while the remaining ones were kept fixed.

One important aspect was the influence of the measured observable on the dynamics of the model. We identified clear differences in the behavior of the χ-capacity depending on the choice of the observable subject to continuous measurement. This dependence suggests that different types of environmental coupling, sensitive to different aspects of the underlying dynamics, may lead to qualitatively distinct transport regimes.

In particular, we identified regimes in which the χ-capacity exhibits asymptotic decay, as well as regimes in which it remains at a non-negligible level over relatively long observation times, exceeding the characteristic timescales of the unobserved dynamics by two orders of magnitude. These results indicate that the effects of continuous measurement may significantly influence the dynamics in regimes where coherent effects become relevant within the model. From an effective-theory standpoint, continuous nonselective measurement may be interpreted as a description of environmental interactions, such as protein fluctuations or solvent dynamics, with the parameter σ characterizing their effective strength.

It should be noted that, although a quantum description may prove redundant for many biological problems, the study of systems at the nanoscale, i.e., those whose dimensions approach the molecular scale, can motivate the use of models accounting for quantum effects, especially in the context of measurement. Moreover, a quantum description can be naturally linked to an information-theoretic framework due to its well established connection with information theory. In this work, ion channels were treated within an effective information-theoretic framework, which may provide an additional perspective for analyzing transport processes in reduced models of biological systems.

At the current stage of research in this very important field of biophysics, the quantum information capacity of ion channels remains primarily a mathematical property of the model used. It should be emphasized that until experiments confirming the applicability of the three-state model studied in this work are demonstrated, it is difficult to determine the extent of universality of the obtained results. However, any modeling that is quantum in nature can, within a certain range of model parameters, lead to the occurrence of deep quantum properties. Our results are primarily an attempt to indicate the extent to which a highly simplified three-state model can indicate the occurrence of such properties in the systems to which it is applied, and therefore, in our case, ion channels.

In this context, it is also natural to ask how the present approach relates to atomistic simulation methods. The relation between the present phenomenological model and atomistic approaches, such as molecular dynamics or molecular mechanics simulations, is not straightforward, as these methods operate at different levels of description. While atomistic simulations resolve detailed interactions within the protein environment, solvent, and ion coordination, the effective model employed here captures the dynamics of the selectivity filter in terms of a small number of dominant configurations and their transitions. In this sense, the two approaches may be viewed as complementary. The effective states considered in this work may correspond to long-lived or recurrent configurations observed in molecular simulations, while the structure of the Hamiltonian reflects preferred pathways of ion transport. This suggests that the present framework can provide a conceptual link between microscopic simulations and more abstract, information-based descriptions of transport. The incorporation of quantum-information measures, such as the χ-capacity, into atomistic simulation frameworks remains an open problem for future research.

## Data Availability

Data is contained within the article.

## References

[1] Roux B., Allen T., Berneche S., Im W. (2004). Theoretical and computational models of biological ion channels. Q. Rev. Biophys..

[2] Kuyucak S., Bastug T. (2003). Physics of ion channels. J. Biol. Phys..

[3] Dixit P.D., Asthagiri D. (2011). Thermodynamics of ion selectivity in the KcsA K^+^ channel. J. Gen. Physiol..

[4] Luchinsky D.G., Gibby W.A., Kaufman I., Timucin D., McClintock P. (2016). Statistical theory of selectivity and conductivity in biological channels. arXiv.

[5] Kaufman I.K., McClintock P.V., Eisenberg R. (2015). Coulomb blockade model of permeation and selectivity in biological ion channels. New J. Phys..

[6] Gibby W., Barabash M., Guardiani C., Luchinsky D., McClintock P. (2021). Physics of selective conduction and point mutation in biological ion channels. Phys. Rev. Lett..

[7] Xie D., Chao Z. (2023). A Poisson-Nernst-Planck single ion channel model and its effective finite element solver. J. Comput. Phys..

[8] Liu J.L., Eisenberg B. (2020). Molecular mean-field theory of ionic solutions: A Poisson-Nernst-Planck-Bikerman model. Entropy.

[9] Aryal P., Sansom M.S., Tucker S.J. (2015). Hydrophobic gating in ion channels. J. Mol. Biol..

[10] Luchinsky D.G., McClintock P.V. (2021). Introduction to the physics of ionic conduction in narrow biological and artificial channels. Entropy.

[11] Cannon R.C., D’Alessandro G. (2006). The ion channel inverse problem: Neuroinformatics meets biophysics. PLoS Comput. Biol..

[12] Kariev A.M., Green M.E. (2021). Quantum calculations on ion channels: Why are they more useful than classical calculations, and for which processes are they essential?. Symmetry.

[13] Gulden T., Kamenev A. (2021). Dynamics of Ion Channels via Non-Hermitian Quantum Mechanics. Entropy.

[14] Salari V., Naeij H., Shafiee A. (2017). Quantum interference and selectivity through biological ion channels. Sci. Rep..

[15] Vaziri A., Plenio M.B. (2010). Quantum coherence in ion channels: Resonances, transport and verification. New J. Phys..

[16] Seifi M., Soltanmanesh A., Shafiee A. (2022). Quantum coherence on selectivity and transport of ion channels. Sci. Rep..

[17] Matulef K., Annen A.W., Nix J.C., Valiyaveetil F.I. (2016). Individual ion binding sites in the K+ channel play distinct roles in C-type inactivation and in recovery from inactivation. Structure.

[18] Holevo A.S. (1973). Bounds for the quantity of information transmitted by a quantum communication channel. Probl. Peredachi Informatsii.

[19] Mironenko A., de Groot B.L., Kopec W. (2024). Selectivity filter mutations shift ion permeation mechanism in potassium channels. PNAS Nexus.

[20] Polakowski M., Panfil M. (2025). Quantum features of the transport through ion channels in the soft knock-on model. Phys. Biol..

[21] Jacobs K., Steck D.A. (2006). A straightforward introduction to continuous quantum measurement. Contemp. Phys..

[22] Jacobs K. (1999). Quantum Measurement Theory and Its Applications.

[23] Breuer H.P., Petruccione F. (2003). The Theory od Open Quantum Systems.

[24] Alicki R., Lendi K. (2007). Quantum Dynamical Semigroups and Applications.

[25] Holevo A.S. (2019). Quantum Systems, Channels, Information.

[26] Lambert N., Gigu’ere E., Menczel P., Li B., Hopf P., Su’arez G., Gali M., Lishman J., Gadhvi R., Agarwal R. (2026). QuTiP 5: The Quantum Toolbox in Python. Phys. Rep..

[27] Johansson J., Nation P., Nori F. (2012). QuTiP: An open-source Python framework for the dynamics of open quantum systems. Comput. Phys. Commun..

[28] Johansson J., Nation P., Nori F. (2013). QuTiP 2: A Python framework for the dynamics of open quantum systems. Comput. Phys. Commun..

